# Possible adsorption mechanism of high mobility group box 1 protein on a polyacrylonitrile (AN69ST) membrane filter

**DOI:** 10.1186/cc10986

**Published:** 2012-03-20

**Authors:** O Nishida, M Yumoto, K Moriyama, Y Shimomura, T Miyasho, S Yamada

**Affiliations:** 1 Fujita Health University School of Medicine, Toyoake, Japan; 2 Rakuno Gakuen University, Ebetsu, Japan; 3 Shino-Test Corporation, Sagamihara, Japan

## Introduction

At ISICEM 2011, we reported that AN69ST showed the highest capacity to adsorb high mobility group box 1 protein (HMGB1) when compared with polymethylmethacrylate, polysulfone and high cut-off membrane [1]. Here we focus on whether filtration or surface heparin on AN69ST by a priming circuit with a heparinized saline contributes to HMGB1, with a heparin-binding protein, adsorption on AN69ST.

## Methods

The test solution contained 100 g HMGB1 and 35 g albumin in 1,000 ml substitution fluid. We executed three different experimental hemofiltrations with solution flow of 100 ml/minute and: ultrafiltrate flow 1,000 ml/hour using AN69ST primed with a heparinized saline, F(+) and H(+); ultrafiltrate flow 1,000 ml/hour using AN69ST with no heparinized saline, F(-) and H(+); and ultrafiltrate flow of 0 ml/hour using AN69ST with no heparinized saline, F(-) and H(-). In addition, AN69ST membrane was immunostained using an antibody that confirmed dying on human kidney tissue.

## Results

**Figure 1:**
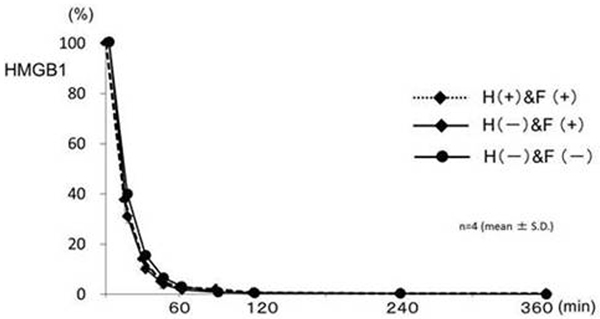
**Time course of HMGB1 levels in the test solution**.

**Figure 2:**
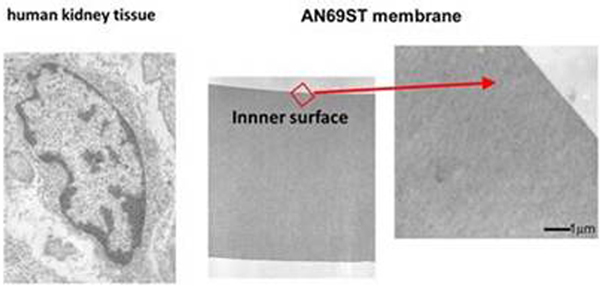
**Immunoelectron microscopy using anti-HMGB1 polyclonal antibodies**.

The concentration decreases of HMGB1 at 0, 60 and 360 minutes indicated no significant differences among the three different hemofiltration experiments (Figure 1). At 60 minutes, reduction rates of HMGB1 were: F(+) and H(+), 97.3%; F(+) and H(-), 94.8%; and F(-) and H(-), 96.4% respectively. HMGB1 was not detected in bulk layers by immunostaining (Figure 2).

## Conclusion

Surface heparin or filtration might not contribute to HMGB1 adsorption on the AN69ST membrane. Remarkable adsorption on AN69ST is likely to be influenced by material characteristics, hydrogel structure with moisture content, or negative electric charge and may occur not in bulk layers but on large surfaces of membranes.
